# Functionalized Polymeric Materials with Bio-Derived Antimicrobial Peptides for “Active” Packaging

**DOI:** 10.3390/ijms20030601

**Published:** 2019-01-30

**Authors:** Bruna Agrillo, Marco Balestrieri, Marta Gogliettino, Gianna Palmieri, Rosalba Moretta, Yolande T.R. Proroga, Ilaria Rea, Alessandra Cornacchia, Federico Capuano, Giorgio Smaldone, Luca De Stefano

**Affiliations:** 1 Materias S.r.l., Corso N. Protopisani, 80146 Napoli, Italy; bruna.agrillo@ibbr.cnr.it; 2 Institute of Biosciences and BioResources, National Research Council (CNR-IBBR), 80131 Napoli, Italy; marco.balestrieri@ibbr.cnr.it (M.B.); marta.gogliettino@ibbr.cnr.it (M.G.); 3 Institute for Microelectronics and Microsystems, National Research Council (CNR-IMM), 80131 Napoli, Italy; rosalba.moretta@na.imm.cnr.it (R.M.); ilaria.rea@na.imm.cnr.it (I.R.); luca.destefano@na.imm.cnr.it (L.D.S.); 4 Department of Food Microbiology, Istituto Zooprofilattico Sperimentale del Mezzogiorno, 80055 Portici, Italy; proroga.yolande@izsmportici.it (Y.T.R.P.); federico.capuano@cert.izsmportici.it (F.C.); 5 National Reference Laboratory for Listeria monocytogenes, Istituto Zooprofilattico Sperimentale dell’Abruzzo e del Molise, 64100 Teramo, Italy; a.cornacchia@izs.it; 6 Department of Agricultural Science, University of Naples “Federico II”, 80055 Portici, Italy; giorgio.smaldone@unina.it

**Keywords:** active packaging, antimicrobial peptides, food shelf-life, foodborne pathogens, plastic materials

## Abstract

Food packaging is not only a simple protective barrier, but a real “active” component, which is expected to preserve food quality, safety and shelf-life. Therefore, the materials used for packaging production should show peculiar features and properties. Specifically, antimicrobial packaging has recently gained great attention with respect to both social and economic impacts. In this paper, the results obtained by using a polymer material functionalized by a small synthetic peptide as “active” packaging are reported. The surface of Polyethylene Terephthalate (PET), one of the most commonly used plastic materials in food packaging, was plasma-activated and covalently bio-conjugated to a bactenecin-derivative peptide named 1018K6, previously characterized in terms of antimicrobial and antibiofilm activities. The immobilization of the peptide occurred at a high yield and no release was observed under different environmental conditions. Moreover, preliminary data clearly demonstrated that the “active” packaging was able to significantly reduce the total bacterial count together with yeast and mold spoilage in food-dairy products. Finally, the functionalized-PET polymer showed stronger efficiency in inhibiting biofilm growth, using a *Listeria monocytogenes* strain isolated from food products. The use of these “active” materials would greatly decrease the risk of pathogen development and increase the shelf-life in the food industry, showing a real potential against a panel of microorganisms upon exposure to fresh and stored products, high chemical stability and re-use possibility.

## 1. Introduction

Today, food preservation, quality maintenance and safety are considered the major growing concerns in the food industry. Food products can undergo different processes of contamination, which lead to loss of colour, texture and nutritive values, allowing the growth of pathogenic microorganisms and deterioration of the quality of the products, making them non-edible. Food contamination can occur with its exposure to the environment during slaughtering, food processing, packaging, transportation or distribution. In addition, one of the main problems in the food industry is represented by the presence of biofilms, which are considered a serious public health risk. A biofilm is a functional consortium of microorganisms formed principally by exopolysaccharides that can exist on all types of surfaces in food plants ranging from plastic, glass, metal, wood, to food products. For these reasons, biofilms enhance the persistence of several foodborne pathogens on product contact surfaces due to their special structure, and so they are more resistant to antimicrobial agents. Current conventional methods for maintaining food quality and safety over time during drying, freezing, heating or salting have not found to satisfy consumers as recontamination may often occur, rendering the food unpalatable.

Another relevant issue concerning the food industry is the need to feed an ever-increasing global population, which makes it obligatory to reduce the millions of tons of avoidable perishable waste along the food supply chain. In this context, a considerable share of these losses is caused by non-optimal chain processes and management. Shelf-life is defined as the time span under defined storage conditions within which foods remain acceptable for human consumption in terms of safety, nutritional attributes and sensory properties [1]. Unappealing foods and the uncertain safety of food items have been reported as the main causes for discarding food products among consumers and retailers. Indeed, about 15% of perishable foods are actually wasted at retail stores due to damage and spoilage [2]. Consequently, prolonging the shelf-life of food products, ensuring their quality, safety, and integrity, is a crucial aspect to minimize food waste. 

All these concerns demand a need for more effective food quality systems for food protection, preservation, and transport to consumers in a wholesome form. Therefore, today, the food industry is more interested in exploring innovative and alternative solutions to presently used methods. In this context, antimicrobial packaging represents a novel strategy to suppress the activities of targeted microorganisms that can contaminate the food products and then strongly affect their shelf-life. One strategy to achieve this goal is to use active materials projected “*ad hoc*” to kill harmful microorganisms or to inhibit their growth on their surface or in the surrounding environments. In this respect, antimicrobial polymers present several advantages because of their high tunability in terms of physico-chemical properties, efficacy, resistance, and prolonged lifetime. However, in spite of the large developments in the preparation and structure-property relationship of this class of antimicrobial polymers, very few of them are practically suitable to solve food-related problems [3]. In this context, plastics are the most commonly used materials for packaging applications because of low-cost, ease of processing and the availability of abundant resources for their production. Indeed, during the last few years, several studies have been focused on the incorporation of antimicrobial peptides (AMPs) into polymeric materials through covalent or physical binding [4,5]. AMPs are essential components of innate immunity [6], contributing to the first line of defence against infections [7,8] and are actually the most promising antimicrobial compounds, mainly because of their broad spectrum of action, high selectivity toward bacterial cells and low risk to promote resistance. The AMP family comprises peptides, which are usually short and amphipathic molecules with a high number of basic residues and a strong tendency to assume prevalently ɑ-helix conformations, which are important to explicate their antimicrobial functions including also antibiofilm activity [9,10,11,12]. Amphiphilic AMPs with net positive charge have the capacity to tune their secondary structure upon interacting with the lipid tails inside the membrane, enhancing the membrane rupture activity of these peptides [13]. One of the most studied AMPs, is the innate defence regulator peptide-1018 (IDR-1018), a 12-mer cationic compound (VRLIVAVRIWRR-NH2), derived from the bovine host-defense peptide (HDP) bactenecin, found in the bovine neutrophil granules and belonging to the cathelicidin family [10,11,12,14,15]. Recently, a new 1018-derivative antimicrobial peptide, named 1018K6, in which the alanine is replaced with a lysine residue (VRLIV**K**VRIWRR-NH2), was designed and characterized [16,17]. This single point mutation was revealed to have a strong impact on the conformational status of 1018K6, inducing an increased propensity to assume an α-helix structure in the membrane-mimetic models such as micellar solutions of SDS [16,17]. Furthermore, 1018K6 was revealed to be able to retain its structural integrity better than the cognate IDR-1018 under a wide range of pH and temperature conditions for prolonged incubation times. In addition, 1018K6 exhibited a significant bactericidal/antibiofilm activity specifically against *L. monocytogenes* isolates from food-products and food-processing environments [16,17]. 

Actually, wet and dry procedures can be used to link peptides to polymer surfaces [18,19], although it is not trivial to functionalize them with AMPs as they can completely lose their antimicrobial activity, once bound on the surface. Cold plasma is considered an emerging novel technology industrially used for activation of polymer surfaces, which exhibit reactive -COOH* and -OH* groups that rapidly interact with the free –NH_2_ and –COOH in the peptide sequence. The resulting functionalized surfaces are very stable and can be used in solution under a wide range of pHs and salt conditions [20].

The aim of this study was to develop a new class of packaging materials, functionalized with the bactericidal peptide 1018K6 by cold plasma technology, able to inhibit the biofilm formation of *L. monocytogenes* and to significantly reduce the Aerobic Plate Count (APC) and yeast and mold spoilage of food dairy products.

## 2. Results and Discussion

### 2.1. Activation of PET Polymer by 1018K6 

Currently, the packaging sector accounts for over 40% of the total worldwide plastic consumption [21,22]. The essential properties for packaging materials are determined by the physical and chemical characteristics of the products, as well as by the external conditions under which the product is stored/transported [21]. As plastics have a wide range of properties which can be tailored according to the specific requirements, they are the most attractive materials for packaging applications. 

**Figure 1:**
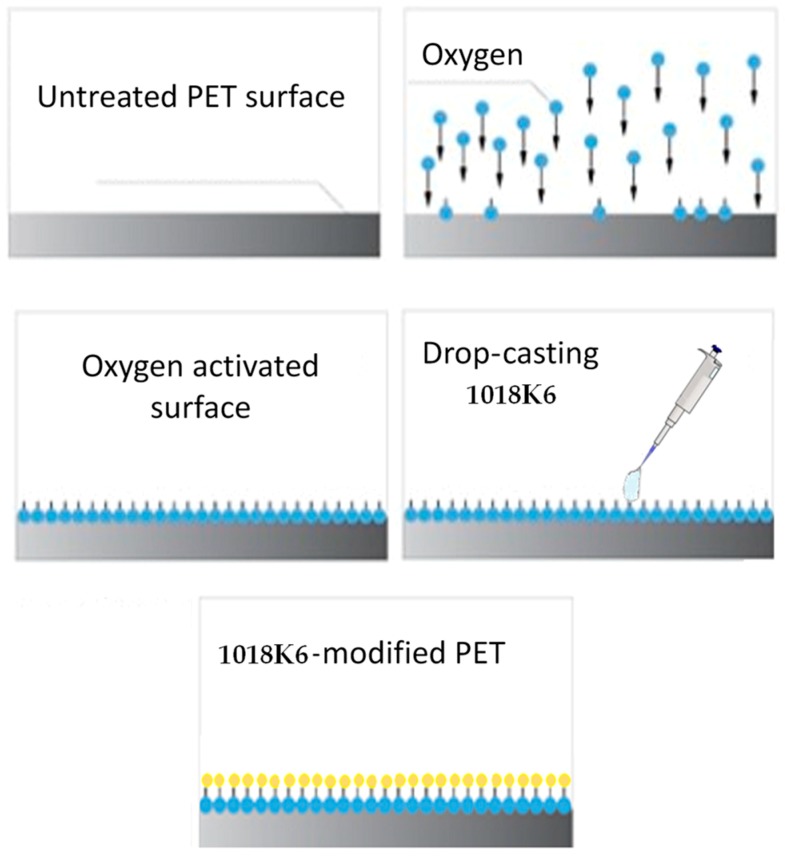
Process diagram for the modification of the PET surface by activation with radiofrequency cold plasma using oxygen as gas and coupling of the synthetic peptide 1018K6 with antibacterial properties.

In this work, polyethylene terephthalate (PET), one of the most common packaging materials accounting for more than 90% of the total volume of plastics used, was functionalized with the already characterized AMP, 1018K6. As the PET surfaces appeared to be hydrophobic, i.e., water contact angle greater than 90°, it was impossible to perform the functionalization by incubating them with the antibacterial peptide 1018K6 in aqueous solutions. Therefore, a possible approach was to pre-activate the PET surfaces by using the radio frequency cold plasma technique and oxygen as gas, which induces the formation of reactive -COOH* and -OH* groups [23], allowing the covalent binding with the peptide, as sketched in Figure 1. 

**Figure 2:**
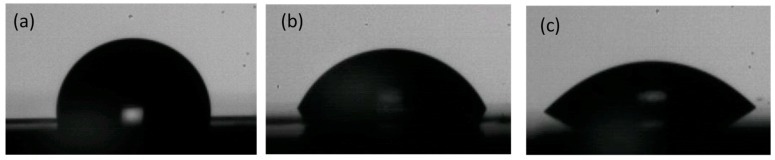
WCA measured on pristine PET (**a**), oxygen plasma activated-PET (**b**) and 1018K6-functionalized PET (**c**). The measurements were performed on five samples in duplicate.

In this experimental procedure, PET disks were taken directly from the container, used for the preservation of fresh dairy products, specifically buffalo mozzarella cheese, and provided by the customer. To assess quantitatively the PET surface wettability induced by oxygen plasma activation and peptide functionalization, WCA (water contact angle) measurements were carried out on PET samples acquiring the images after 30 sec, before and after treatments (Figure 2). 

Firstly, the WCA value of the pristine PET disks was equal to 98 ± 3° (Figure 2a). Upon oxygen plasma treatment, the WCA profiles of PET membranes shifted to lower values, indicating that a higher degree of surface hydrophilicity was achieved. Specifically, the change in the surface wettability was quantified, carrying out the measurements at different exposure times (T) and RF powers and varying the concentration and the partial pressure (P) of the oxygen (O_2_). The obtained results demonstrated that, already at 50 W and 10 sec of exposure time, the PET surface became less hydrophobic and more hydrophilic (WCA = 56 ± 5°), as shown in Figure 2b. However, no variations were observed in WCA values by changing the oxygen pressure and concentration parameters. On the contrary, it should be noted that when high values of RF power (RF = 300 W) and long exposure times (T = 100–300 sec) were applied, a macroscopic change in the roughness of the PET surface was detectable, indicative of the beginning of a material degradation process. This behaviour suggested that the cold radio frequency plasma treatment was not suitable for PET materials under the aforementioned operating conditions. Generally, the surface of the pristine PET was hydrophobic due to the presence of aliphatic carbonaceous chains. Hence, the plasma treatment induced the formation of extremely reactive radical groups that interrupted the carbon chains and reduced the inborn hydrophobicity of the material, making it more hydrophilic and able to interact strongly with water molecules [24]. Immediately after plasma exposure, the pre-treated PET samples were incubated for a minimum of 8 h in an aqueous solution of 1018K6 peptide, using samples not subjected to radio frequency cold plasma treatment as controls. The PET exposure to the peptide solution favoured the coupling between the peptide chemical groups (typically -COOH and –NH_2_) and the generated reactive groups (-COOH*, -OH*) on PET, which were not passivated by the atmospheric water. The coupling of the peptide on the polymeric surface resulted in a further modification of the wettability as revealed by the WCA value (WCA = 36 ± 2°), due to the hydrophilic nature of the chemical groups of the amino acid residues along the peptide sequence (Figure 2c). On the other hand, PET control samples not pre-treated by radio frequency cold plasma and incubated for 24 h in aqueous solution containing 1018K6, clearly showed a negligible non-specific adsorption of the peptide on the PET surface.

**Figure 3:**
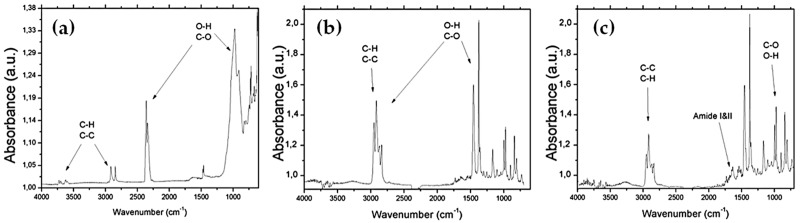
FTIR spectrum of the PET sample before radiofrequency cold plasma treatment (**a**); after plasma treatment (**b**); after 1018K6 bio-conjugation (**c**).

In order to confirm the 1018K6-PET linkage, the Fourier Transform InfraRed spectroscopy (FTIR) was carried out under inert (N2) atmosphere. The FTIR spectra of the control samples before radio frequency cold plasma treatment displayed different main peaks corresponding to the C-C, C-H, C-O groups of the polymer and to the –OH groups of the water adsorbed on the polymer surface after the incubations (Figure 3a).

After the plasma treatment, a relevant increase of the –OH group peaks in the FTIR spectra was observed (Figure 3b), consistent with the improvement of the surface wettability quantified by WCA measurements [25,26]. Next, the functionalization of the activated PET samples with 1018K6 was responsible for the appearance, in the FTIR spectra, of the characteristic absorption signals of a peptide, including the Amide I and Amide II bands (Figure 3c). These bands arise from the peptide bonds that link the amino acids (O=C-NH) in the 1018K6 sequence. Specifically, the absorption associated with the Amide I band, which was observed in the 1650–1560 cm^−1^ interval, produced the stretching vibrations of the C=O bond of the amide, whilst the absorption associated with the Amide II band showed in the 1580–1490 cm^−1^ interval, led primarily to bending vibrations of the N—H bond (Figure 3c). Therefore, the FTIR analyses validated the successful bio-conjugation of 1018K6 peptide on the plasma-activated PET surface, in complete agreement with WCA characterization.

### 2.2. Immobilization Yield and Leakage of 1018K6 from PET Polymer 

**Figure 4:**
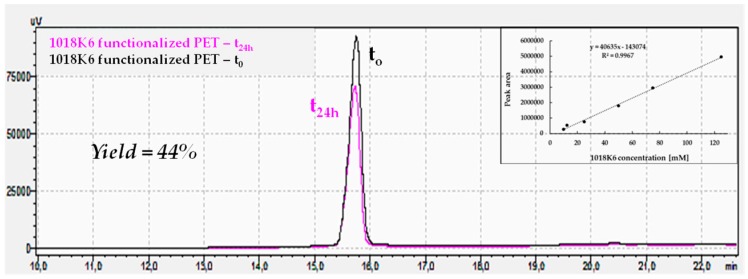
Immobilization yield (%) of 1018K6 on PET surface determined by reverse-phase HPLC chromatography on a C18 column after the coulping reaction (24 h). Pre-activated PET surfaces by plasma were incubated for 24 h with 1018K6 (50 µM) in PBS pH 7.0. The solutions recovered after incubation were further analysed. The peptide solution placed in contact with the pre-activated surface at time 0 (*t* = 0) was used as control. The chromatograms are representative of three independent experiments. **Insert**: Calibration curve of the C18 column obtained using different 1018K6 concentrations.

**Figure 5:**
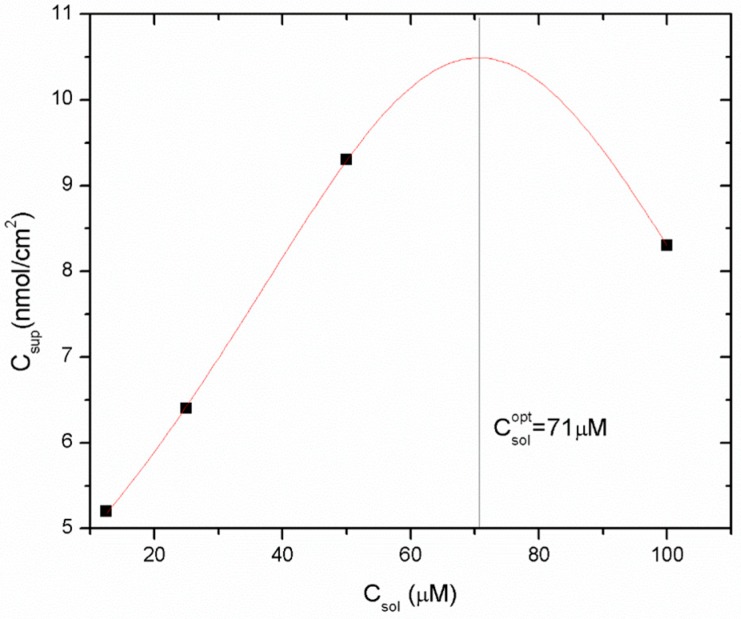
Immobilization yield expressed as nmol of bound 1018K6 per cm^2^ of PET surface as function of peptide concentration. The dose-response curve has been built by using the Holliday model. Data are expressed as means ± standard deviations. Standard deviation values lower than 5% are not shown.

One of the most important factors in fabricating antimicrobial packaging is to immobilize on a polymeric surface the functional compounds without losing their activity. Therefore, to keep them active, it is necessary to immobilize the peptides in a way that preserves their folded structural integrity. Firstly, to obtain stable and active packaging, it is crucial to regulate the peptide surface concentration which depends on the binding strategy used, as it can strongly affect the efficiency of peptide immobilization. Therefore, the immobilization yield of different 1018K6 concentrations on the PET surface after the coupling reaction was indirectly estimated by Reverse-Phase High-Performance Liquid Chromatography (RP-HPLC). In this experiment, once the conjugation reaction was completed, the supernatant solutions were recovered after 24 h incubation and analysed by RP-HPLC, evaluating the peak area of the peptide not bound to the polimeric surface. Consequently, by knowing the initial peptide concentration, the quantity of the peptide attached to the PET surface was indirectly determined by comparing the peak area. The data obtained from these analyses showed that the coupling reaction yield varied from 50% using a starting peptide concentration of 25 µM, to 25% per 100 µM. The representative chromatograms obtained for 1018K6 50 µM initial concentration, and used to calculate the immobilization yield, are reported in Figure 4. The coupling yields were validated by a six-point calibration curve, which was constructed utilizing known 1018K6 concentrations, and the number of peptide molecules capable of binding to the polymeric surface was determined via interpolation (Figure 4 insert). Based on the yield data, the surface coverage on the polymer was found to be approximately 6.4 nmol/cm^2^ per 25 µM peptide concentration, 9.3 nmol/cm^2^ per 50 µM and 8.3 nmol/cm^2^ per 100 µM, thus demonstrating that the surface coverage was clearly concentration-dependent (Figure 5). 

The Holliday model was used to assess the peptide concentration effects on the coverage density and to estimate the concentration value producing the best immobilization yield [27]. As shown in the dose-response experiments (Figure 5), the most suitable coupling condition to improve the immobilization yield was obtained with a peptide concentration of 71 µM, but 50 μM was selected to perform the further experiments as this value represents a better compromise between the functionalization yield and the peptide costs.

The production process of antimicrobial active packaging, which is able to guarantee the quality, the safety and prolong the shelf-life of food products, requires an efficient immobilization procedure that permits a stable conjugation of AMP on the polymers, avoiding the release of the immobilized active compound after contact with foods or liquids. Fresh dairy products, such as mozzarella cheese, an Italian traditional cheese packaged in saline brine, are ready-to-eat foods having a very short shelf-life of about 3 or 4 days, because they are easily contaminated by undesirable microorganisms. 

**Figure 6:**
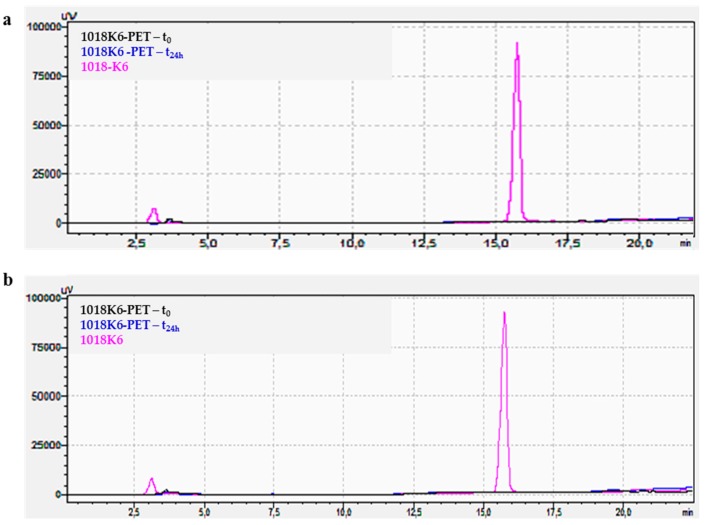
Release analysis of 1018K6 from functionalized PET performed by reverse-phase HPLC chromatography on a C18 column after 24 h incubation at 4 °C in mozzarella brine (**a**) or pure water (**b**). After incubation, the solutions were recovered and injected on C18. The solution in contact with 1018K6-PET at time 0 (*t* = 0) and 1018K6 peptide (50 μM) were used as controls.

In this context, the release of 1018K6 from the functionalized PET into mozzarella cheese brine during 24 h of incubation at 4 °C was analysed by RP-HPLC, using the free 1018K6 as control. As shown in Figure 6a, no peptide-release process occurred from the functionalized polymeric support. The same results were obtained after 24 h incubation in pure water at 4 °C (Figure 6b) and at 25 °C suggesting that the peptide was stably coupled on the polymer. In addition, no leakage of 1018K6 was detectable even after prolonged incubations (until to 72 h) under all the conditions explored. The high stability of the peptide-PET bond is important because in this way the peptide-PET system does not require the related EFSA (European Food Safety Authority) standards. 

However, as far as the cytotoxicity of the free 1018K6, preliminary tests clearly indicated that the peptide is not toxic against different fibroblast cell lines at the concentrations used in the bactericidal assays [16], thus suggesting that there is no potential risk for human health associated with the use of 1018K6 in the food industry. 

### 2.3. Effect of 1018K6 Functionalized PETs on Mozzarella Cheese

Microbial contamination, causing approximately one-fourth of the world’s food supply loss, has become an enormous economic and ethical problem worldwide [28]. Specifically, fresh dairy products stored in packaging, such as mozzarella cheese, are characterized by reduced shelf-life, which diminishes their commercial value because they are an excellent growth medium for a wide range of troublesome spoilage microorganisms including aerobic mesophiles, yeasts and molds [29]. 

Hence, it is very important and advantageous for the food industry to extend the shelf-life of mozzarella cheese, which is a good source of protein, vitamins and minerals, and to spread the distribution of this traditional product beyond the market borders. 

In this context, the efficacy of 1018K6 functionalized PETs in preventing the growth of spoilage microorganisms in mozzarella was analysed at a preliminary shorter storage time. In a first set of experiments, with the aim to set up the optimal experimental conditions minimizing peptide consumption, PET disks of 3 cm diameter (surface of 7 cm^2^) were functionalized with 1018K6 and incubated with 3 ml of the conditioning brine in the presence of small slices of mozzarella (about 10 g of weight) in Petri dishes. Sliced mozzarella in the presence of brine with non-modified PETs disks was used as controls. As shown in Table 1, the Aerobic Plate Count (APC), and the yeast and mold counts of the samples exposed to 1018K6-PETs significantly decreased during the storage period (24 h) compared to the control samples. In a second set of experiments, a scale up of the previous procedure was applied in order to evaluate the effectiveness of 1018K6-PETs in slowing down the growth of the spoilage microorganisms under the storage conditions. Specifically, the effects of 1018K6-PET disks (10 cm diameter, 78 cm^2^) were studied directly in the package as distributed on the market, which contained two balls of fresh mozzarella (about 25 g each) and 30 mL brine. Control samples were prepared in an identical way, using non-modified PET disks. Results demonstrated that, in one day of storage, mozzarella packaged in the presence of 1018K6-PETs had the lowest bacterial counts with respect to that incubated in conditioning brine with non-modified PETs, in which microbes were able to proliferate (Table 1). In addition, a significant reduction in yeasts and molds count was also observed in the samples with the modified PETs during the storage. Therefore, the projected 1018K6 active packaging could have potential applications in the food market, aiming to ensure and increase the quality and safety of the food products by preventing the growth of spoilage and/or pathogenic microorganisms and promoting a shelf-life extension. 

### 2.4. Inhibition of Listeria Biofilm Formation 

Cross-contamination of pathogenic and spoilage microorganisms from food contact surfaces remains a significant challenge in the safety, quality and security of food supply chain. Indeed, some pathogenic and food spoilage bacteria can form biofilms, which represent one of the main sources of food contamination and foodborne disease outbreaks. To address this challenge, there is an unmet need to develop novel antimicrobial materials able to inhibit and treat biofilms in the food processing industry. Specifically, the use of natural preservatives to inhibit growth of serious pathogens such as *L. monocytogenes* is of great interest as it is considered an important worldwide public health problem [30]. *L. monocytogenes* is one of the most dangerous human food pathogens that causes listeriosis. Foods considered as high-risk sources of listeriosis include meat and dairy products, which are ready-to-eat, require refrigeration and are stored for extended time periods. Listeria can persist within food processing environments, due to its ability to grow at wide-ranging temperatures and pH and to form biofilms [31,32]. 

**Figure 7:**
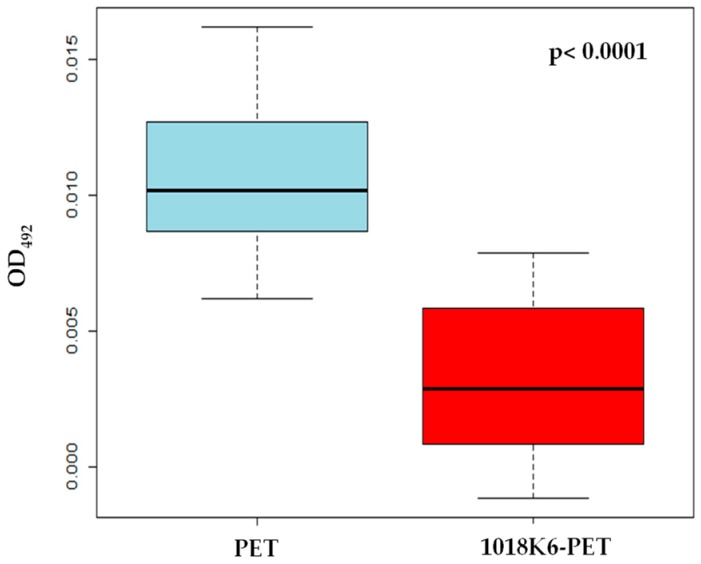
Boxplot of the inhibition activity of the biofilm production of *Listeria monocytogenes* by PET and 1018K6-PET. Average OD measurements of crystal violet-stained biofilms are shown with error bars representing the standard deviation.

In this context, the ability of 1018K6-PETs to prevent biofilm formation was assessed against an *L. monocytogenes* strain isolated from dairy products, by the crystal violet staining method [17]. As shown in Figure 7, the biofilm formation on 1018K6-PETs was significantly reduced (75%), compared to the control sample (non-functionalized PETs), indicating a strong anti-adhesion capability of the 1018K6-tethered surfaces against *L. monocytogenes*. 

Therefore, packaging films containing 1018K6 peptide can pose a potential solution to reduce spoilage. 

## 3. Materials and Methods

### 3.1. Plasma Treatment

Plasma treatment was performed using a Reactive Ion Etching (RIE) model PLASMA Plus 80 machine (Oxford Instruments, Abingdon, Oxfordshire, UK). The following process parameters were changed: exposure time [T] (10-20-30-50-100-300 sec); molecular oxygen concentration [O2] (10-50-100 sccm); partial gas pressure [P] (0.1–0.5 atm); power of radio frequency generator [RF] (50-100-300 W).

### 3.2. Water Contact Angle Measurements 

The sessile drop technique was used for water contact angle (WCA) measurements on a First Ten Angstroms FTA 1000 C Class coupled with drop shape analysis software under static conditions. A 10-μL drop was deposited on the sample surface, and the image was recorded after 30 sec. Results of WCA are expressed as mean ± standard deviation (s.d.) of at least three measurements on the same sample in three independent experiments (i.e., at least nine measurements for each result).

### 3.3. Fourier Transform Infrared Spectroscopy

The Fourier transform infrared spectra of all samples were obtained using a Nicolet Continuum XL (Thermo Scientific, Waltham, MA, USA) microscope in the wavenumber region of 4000−650 cm^−1^ with a resolution of 4 cm^−1^. The FITR measurements were performed using a micro-ATR (Attenuated Total Reflection) module under inert (N_2_) atmosphere.

### 3.4. Peptide Bio-conjugation

Polymer samples treated by cold plasma were incubated in aqueous solution of 1018K6 (50 µM) in PBS (10 mM), pH 7.0, for 24 h at 25 °C. After incubation, the solutions containing the peptide not bound to the polymer were removed and the functionalized PETs were extensively washed in water and DMSO in order to completely eliminate traces of unbound peptide before performing surface characterization by WCA, FTIR and the release experiments. The PET containers used in all the analyses were kindly provided by the dairy “Mini Caseificio Costanzo s.r.l.” located in Lusciano (Caserta, Italy). 

### 3.5. Functionalization Yield Analysis of Polymers

Functionalization yield analysis of 1018K6-modified PETs was performed by using a reverse-phase high-performance liquid chromatography (RP-HPLC) system (Waldbronn, Germany). Once functionalization was completed, the supernatant solutions were recovered after 24 h and analyzed to calculate the amount of the peptide not attached to the polymeric surfaces. For the analyses, 200 μL of the samples were injected over a μBondapak C18 reverse-phase column (3.9 mm × 300 mm, Waters Corp., Milford, MA, USA) connected to a HPLC system (Shimadzu, Milan, Italy) using a linear gradient of 0.1% TFA in acetonitrile from 5 to 95%. A reference solution was prepared with the initial peptide concentration used for the functionalization under the same reaction conditions and run in parallel. Therefore, by knowing the added peptide (reference solution), the amount of peptide not bound to the polymers (expressed as a percentage) was determined by comparing the peak area. A calibration curve of the C18 column using different 1018K6 concentrations was built. All measurements were performed in triplicate in three different preparations.

### 3.6. Release Test

The release of 1018K6 from the functionalized polymers was examined by the reverse-phase high-performance liquid chromatography (RP-HPLC) system using a μBondapak C18 column (3.9 × 300 mm, Waters) and a linear gradient of 5–95% acetonitrile in 0.1% TFA, at a flow rate of 1 mL/min. A volume of 1 mL of pure water or mozzarella cheese brine was poured onto the functionalized polymers, incubated for 24 h at 4 °C and then loaded onto the RP column. The solutions in contact with the functionalized polymers at time t = 0 were used as control samples and were run in parallel. The same experiments were conducted in the presence of the non-functionalized polymers. All measurements were performed in triplicate on three different preparations.

### 3.7. Shelf-life Testing on Mozzarella Cheese

The 1018K6-functionalized PETs were cut into disks of 3 cm diameter (surface of 7 cm^2^) and immersed in 3 mL mozzarella cheese brine in 5-cm Petri dishes that contained small slices of mozzarella (about 10 g of weight), which were directly placed on the activated PET disks. Non-functionalized PETs were used as a control. The samples were incubated for 24 h at 25 °C. Therefore, the mozzarella cheese brine was plated on PCA plates to quantify the APC (Aerobic Plate Count), which was performed according to ISO 4833-1 procedure. Specifically, 1 mL of cheese brine was diluted in 9 mL diluent (0.1% peptone and 0.8% sodium chloride, biomerieux- France), and scalar dilutions of sample up to 10^−5^ were set up. Then, 1 mL of brine and 1 mL of each subsequent dilution were seeded by inclusion in PCA plates (Plate Count Agar-Biolife-Italy) which were incubated at 30 ± 1 °C for 72 h. Plates with no more than 300 colonies were considered for the colony count. The presence of yeasts and molds was tested, according to ISO 21527-1, analyzing 1 mL of cheese brine, diluted in 9 mL diluent (0.1% peptone and 0.8% sodium chloride, biomerieux- France) and performing scalar dilutions up to 10^−5^. Then, 0.1 mL of each dilution was seeded on Dichloran Rose Bengal Chloramphenicol Agar plates (DRBC - Italian Biolife), which were incubated at 25 ± 1 °C for 5 days for the colony count. Plates with no more than 150 colonies were considered. The same analyses were performed using 1018K6-PET disks of 10 cm diameter (78 cm^2^), immersed in the package containing two balls of fresh mozzarella (about 25 g each) and 30 mL brine. The samples were incubated for 24 h at 25 °C. The analyses were performed in triplicate on three different preparations, and the data were expressed as means ± s.d.

### 3.8. Anti-adhesion Activity Assay

*L. monocytogenes* cultures, isolated from dairy products, were prepared to inoculate BHI broth (Brain Heart Infusion, Sigma-Aldrich, St. Louis, Missouri, USA) at 37 °C up to a logarithmic phase of growth. After the incubation, 10 ml of bacterial suspension at a concentration of 5 × 10^6^ in growth broths was centrifuged, and the cell pellet was washed in PBS pH 7.3 (Thermo Fisher Scientific Inc., Waltham, MA, USA) and diluted in BHI broth to reach the useful concentration to obtain biofilm formation. The assays were conducted using PET disks as the food contact surface. PETs were placed into 12-well tissue culture plates (Falcon, Thermo Fisher Scientific Inc., Waltham, MA, USA), with a flat bottom and lid. After washing in sterile ultrapure water, the PETs were incubated in ethanol (≥ 99.8%) for 10 min under gentle shaking and were then washed in sterile ultrapure water, dried and packaged. In each experiment set, 600 μL of the standardized inoculum in the presence of 1018K6- PETs or non-functionalized PETs was added to 12-well tissue culture plates. BHI broth was used as negative control, and the plates were incubated at 37 °C for 72 h under the static condition. Cell counting of *L. monocytogenes*, in agreement with the ISO 11290-2:98 (ISO 11290-2: 1998/Amd 1, 2004) method, was performed to assess the concentration and purity of the standardized inoculum. After incubation, PETs were washed three times with PBS pH 7.3 and placed in a new plate to dry. At the end of the fixing phase, 1 mL of 0.2% Crystal Violet (Panreac Quimica SAU, Barcelona, Spain) in 95% ethanol was added to each well to stain the PETs. After gentle shaking for 15 min, the PETs were washed three times with sterile water and were then transferred into a new plate to dry at 37 °C. The quantitative analysis of biofilm production was performed by adding 1 mL of 33% acetic acid to destain the PETs, and 200 μL of each solution was transferred to a microtiter plate to measure the level (OD492) of the crystal violet. Anti-adhesion assay was performed in triplicate on three independent sets of experiments. OD492 values were compared through non-parametric analysis of variance (Kruskal-Wallis test), followed by multiple comparisons using Dunn test pairs (with Bonferroni correction) (*p* < 0.05). Statistical analyses were performed using Microsoft®Excel 2000/XLSTAT©-Pro.

## 4. Conclusions

Adding new functionalities to food packaging is a key issue in the production of the next generation of active materials. In this context, polymers represent good candidates as their production, use and disposal/recovery are well established at very low costs. Among the main useful packaging materials, PET is one of the most widely employed worldwide in the food industry. 

The results of this study demonstrated that PET material can be efficiently and quickly pre-activated by the cold oxygen plasma technique, which represents an industrial scalable technology, in order to promote the functionalization with 1018K6, a peptide showing potent antibacterial and anti-adhesion properties, and to obtain antimicrobial packaging. 1018K6-PETs were tested under real conditions, using samples of mozzarella cheese, and it was found that APC, yeast and mold counts of the samples stored in the presence of modified polymers were strongly reduced during the first 24 h, thus demonstrating the 1018K6 was still active and preserved its antimicrobial abilities upon polymer surface immobilization. Moreover, 1018K6-PET was very effective against the formation of *Listeria* biofilms, a non-trivial result since not all antimicrobial agents are able to combat bacterial biofilms.

This work represents a preliminary study, which provides a starting point to develop a new PET-based system, functionalized with a biologically-derived AMP, which can have potential as antimicrobial packaging, providing an innovative and breakthrough technology in food applications, due to its comparable cost, small peptide dimension, effective antimicrobial activity, polymer characteristics and environmental friendliness. However, further investigations will be required to establish whether the projected antimicrobial-polymers may find industrial uses and whether they will be effective to improve the safety and extend the shelf-life of food products.

## 5. Patents

International Patent, Application No: PCT/EP2018/069304 Publication Date: 16/07/2018. Antimicrobial peptides. BALESTRIERI Marco, PALMIERI Gianna, CAPUANO Federico, DE STEFANO Luca et al.

## Figures and Tables

**Table 1:** Effects of 1018K6-PETs treatment on mozzarella cheese.

Disk Diameter	Microorganisms	PET Disk in Brine + Mozzarella Cheese	1018K6-PET Disk in Brine + Mozzarella Cheese	Inhibition of Growth (% Value)
3 cm	APC	311 ± 29 CFU/mL	11 ± 2 CFU/mL	97%
	Yeasts and Molds	700 ± 75 CFU/mL	280 ± 25 CFU/mL	60%
10 cm	APC	173 ± 21 CFU/mL	44 ± 7 CFU/mL	75%
	Yeasts and Molds	406 ± 37 CFU/mL	137 ± 23 CFU/mL	67%

Further studies will be necessary in order to assess the ability of 1018K6-PETs to affect the APC and the total yeast and mold at prolonged storage times.
